# Supplementary material for: Stable memory with unstable synapses
Source: Nat Commun. 2019 Sep 30;10:4441. doi: 10.1038/s41467-019-12306-2 (PMC6768856; doi:10.1038/s41467-019-12306-2)
Supplement: Supplementary file 1 — Supplementary Information [file 41467_2019_12306_MOESM1_ESM.pdf]

Supplementary information:
Stable memory with unstable synapses

Susman et al.

Supplementary Notes

1. A low-dimensional approximation of imaginary-coded memories

In the main text, neural dynamics during retrieval of an embedded memory were shown to converge to a limit-cycle attractor. Here we show that these dynamics can be well approximated by a two-dimensional system.

Consider the neural dynamics presented in Equation (1) in the main text:

$$\dot{\mathbf{x}} = -\mathbf{x} + \mathbf{W}\phi\left(\mathbf{x}\right), \quad \mathbf{x}(t=0) \neq 0 \tag{1}$$

where one memory plane is embedded into the connectivity matrix:

$$\mathbf{W} = \rho\left(\mathbf{u}\mathbf{v}^T - \mathbf{v}\mathbf{u}^T\right) + \gamma\left(\mathbf{u}\mathbf{u}^T + \mathbf{v}\mathbf{v}^T\right),$$

for $\rho > 0,\ \gamma > 1$ and $\mathbf{u}, \mathbf{v} \in \mathbb{R}^N$ with independently drawn, Normally distributed components; the vectors are scaled to have unit norm. Defining the projected coordinates

$$p_{\mathbf{u}} := \mathbf{u}^T\mathbf{x}/\sqrt{N}, \qquad p_{\mathbf{v}} := \mathbf{v}^T\mathbf{x}/\sqrt{N},$$

we have from Supplementary Equation (1)

$$\begin{aligned} \dot{p}_{\mathbf{u}} &= -p_{\mathbf{u}} + \left(\rho\mathbf{v}^T + \gamma\mathbf{u}^T\right)\phi/\sqrt{N} \\ \dot{p}_{\mathbf{v}} &= -p_{\mathbf{v}} + \left(-\rho\mathbf{u}^T + \gamma\mathbf{v}^T\right)\phi/\sqrt{N}. \end{aligned} \tag{2}$$

The Jacobian of Supplementary Equation (1) has a complex conjugate eigenvalue pair with positive real part, and all other eigenvalues have negative real part. Linear stability theory thus predicts that solutions converge onto the $\mathbf{uv}$ plane, so we express $\mathbf{x} = p_{\mathbf{u}}\mathbf{u} + p_{\mathbf{v}}\mathbf{v}$. Now, we approximate the sigmoid ϕ by a step-function:

$$\phi_i\left(x_i\right) = \text{sign}\left[p_{\mathbf{u}}u_i + p_{\mathbf{v}}v_i\right].$$

Using this, we can approximate the coordinates of the projected rate-vector

$$\begin{aligned} q_{\mathbf{v}} := \mathbf{v}^T\phi/\sqrt{N} &\approx \sum_i v_i \text{sign}\left[p_{\mathbf{u}}u_i + p_{\mathbf{v}}v_i\right]/\sqrt{N} \\ &= \sum_i |v_i| \text{sign}\left[p_{\mathbf{u}}\frac{u_i}{v_i} + p_{\mathbf{v}}\right]/\sqrt{N} \\ &= \frac{1}{\sqrt{N}}\sum_i |v_i| s_i; \end{aligned} \tag{3}$$

for convenience, we have defined $s_i := \text{sign}\left[p_{\mathbf{u}}\frac{u_i}{v_i} + p_{\mathbf{v}}\right]$. Using this representation, we wish to express the coordinates $q_{\mathbf{u}},\ q_{\mathbf{v}}$ as functions of the coordinates $p_{\mathbf{u}},\ p_{\mathbf{v}}$. First, by definition

$$s_i = 1 \iff p_{\mathbf{v}} > -\frac{u_i}{v_i}p_{\mathbf{u}}.$$

Second, since $u_i,\ v_i$ are independent Normal random variables, the quotient $a := -\frac{u_i}{v_i}$ has a standard Cauchy distribution, with cumulative distribution function

$$\Pr\left(a \leq x\right) = \frac{1}{\pi}\arctan\left(x\right) + 1/2.$$

Thus,

$$\Pr\left(s_i = 1\right) = \Pr\left(a p_{\mathbf{u}} < p_{\mathbf{v}}\right).$$

Assume first that $p_{\mathbf{u}} > 0$, so

$$\begin{aligned}&\Pr\left(a p_{\mathbf{u}} < p_{\mathbf{v}}\right) = \\ &\Pr\left(a < \frac{p_{\mathbf{v}}}{p_{\mathbf{u}}}\right) = \frac{1}{\pi}\arctan\left(\frac{p_{\mathbf{v}}}{p_{\mathbf{u}}}\right) + 1/2.\end{aligned}$$

For $p_{\mathbf{u}} < 0$ we have

$$\begin{aligned}&\Pr\left(a p_{\mathbf{u}} < p_{\mathbf{v}}\right) = \\ &\Pr\left(\frac{p_{\mathbf{v}}}{p_{\mathbf{u}}} < a\right) = 1 - \Pr\left(\frac{p_{\mathbf{v}}}{p_{\mathbf{u}}} > a\right) \quad = -\frac{1}{\pi}\arctan\left(\frac{p_{\mathbf{v}}}{p_{\mathbf{u}}}\right) + 1/2.\end{aligned}$$

In total, we get

$$\Pr\left(s_i = 1\right) = \frac{\operatorname{sign}\left[p_{\mathbf{u}}\right]}{\pi}\arctan\left(\frac{p_{\mathbf{v}}}{p_{\mathbf{u}}}\right) + 1/2.$$

For $s_i = -1$ we similarly obtain

$$\Pr\left(s_i = -1\right) = \Pr\left(\frac{p_{\mathbf{v}}}{p_{\mathbf{u}}} < a\right) \quad = -\frac{\operatorname{sign}\left[p_{\mathbf{u}}\right]}{\pi}\arctan\left(\frac{p_{\mathbf{v}}}{p_{\mathbf{u}}}\right) + 1/2.$$

The expected value for s_i is then

$$\begin{aligned}\langle s\rangle_{\mathbf{u},\mathbf{v}} &= (+1)\Pr\left(s_i = 1\right) + (-1)\Pr\left(s_i = -1\right) \\ &= \frac{2}{\pi}\operatorname{sign}\left[p_{\mathbf{u}}\right]\arctan\left(\frac{p_{\mathbf{v}}}{p_{\mathbf{u}}}\right).\end{aligned}$$

Inserting this into the sum in Supplementary Equation (3), approximating $|v_i| \approx \sqrt{\frac{2}{N\pi}}$ (the expectation of the absolute value of a Normally distributed variable), and neglecting the correlations between $|v_i|$ and s_i, we obtain

$$\begin{aligned}q_{\mathbf{v}} = \frac{1}{\sqrt{N}}\sum_i |v_i| s_i &\approx \left(\frac{2}{\pi}\right)^{3/2}\operatorname{sign}\left[p_{\mathbf{u}}\right]\arctan\left(\frac{p_{\mathbf{v}}}{p_{\mathbf{u}}}\right) \\ &= \left(\frac{2}{\pi}\right)^{3/2}\arctan\left(\frac{p_{\mathbf{v}}}{|p_{\mathbf{u}}|}\right)\end{aligned}$$

and a similar derivation gives us

$$
\begin{aligned}
q_{\mathbf{u}} &:= u^T \phi / \sqrt{N} \\
&\approx \left(\frac{2}{\pi}\right)^{3/2} \arctan\left(\frac{p_{\mathbf{u}}}{|p_{\mathbf{v}}|}\right)
\end{aligned}
$$

So, inserting the expressions of $q_{\mathbf{u}}, q_{\mathbf{v}}$ into Supplementary Equation (2), we have

$$
\begin{aligned}
\dot{p}_{\mathbf{u}} &= -p_{\mathbf{u}} + \gamma q_{\mathbf{u}} + \rho q_{\mathbf{v}} \\
\dot{p}_{\mathbf{v}} &= -p_{\mathbf{v}} + \gamma q_{\mathbf{v}} - \rho q_{\mathbf{u}}.
\end{aligned} \tag{4}
$$

Numerically simulating the two-dimensional system, we indeed find a stable limit-cycle around the origin (Supplementary Figure 3A). Beyond the qualitative similarity, the approximate model also captures quantitative aspects of the attractor, namely, the radius of the limit cycle over a wide range of parameter values (Supplementary Figure 3 B,C).

2. Capacity of the 'Limit-cycle Hopfield' model

In this section we numerically evaluate the storage capacity of our limit-cycle variant of the Hopfield model, and compare it to the classical, symmetric case [1, 2]. We first discuss the zero-temperature discrete Hopfield model with N neurons and M stored memories [1]:

$$S_i(t+1) = \text{sign}\left[\sum_j W_{ij} S_j(t)\right], \qquad i = 1, ..., N,$$
$$\mathbf{W} = \frac{1}{N}\sum_{k=1}^{M} \mathbf{u}^{(k)} \mathbf{u}^{(k)T}, \tag{5}$$

where the dynamic variable $\mathbf{S}$, as well as the memory patterns $\mathbf{u}^{(k)}$, are binary variables.

In the limit $N, M \to \infty$, the critical memory capacity of the network is $\alpha_c = \lim_{N,M\to\infty} M/N \approx 0.14$ [3]. For $\alpha > \alpha_c$, the overlap of the network state with the target pattern, $m = \mathbf{u}^T\mathbf{S}/N$, sharply declines from 1.

If $\mathbf{W}$ is replaced in Supplementary Equation (5) by any purely anti-symmetric matrix, it can be shown that the dynamics of the state $\mathbf{S}$ always converge onto a stable 4-cycle [4]. In our model, as discussed in the main text, we consider anti-symmetric connectivity matrices of the form

$$\mathbf{W} = \frac{1}{N}\sum_{k=1}^{M}\left(\mathbf{u}^{(k)}\mathbf{v}^{(k)T} - \mathbf{v}^{(k)}\mathbf{u}^{(k)T}\right). \tag{6}$$

The stable cycles arising from this connectivity are precisely $\{\pm\mathbf{u}^{(k)}, \pm\mathbf{v}^{(k)}\}$, thus linking the result of [4] to the geometry of the eigenspace of $\mathbf{W}$.

When assessing the capacity of this model, one must take into account the two-dimensional nature of the attractor states. Thus, we use the L_1 radius on each embedded plane, $m = |q_\mathbf{u}| + |q_\mathbf{v}|$ with $q_\mathbf{u} = \mathbf{u}^T\mathbf{S}/N$, and, for comparison with the symmetric model, a given α is computed for a network embedded with $M/2$ memory planes. In other words, in both cases we are counting the total dimensionality of memory-space.

Supplementary Figure 4A left shows the overlaps obtained from simulating the two discrete variants (Supplementary Equations (5),(6); blue: symmetric Hopfield, and orange: our model, respectively), for a fixed N and varying memory loads α. The critical load is found to be slightly higher for the anti-symmetric model. Varying the network size N, we find that in both models the critical capacity approaches a constant limit - namely, the number of memories that can be embedded in the network scales with N (Supplementary Figure 4A right). Interestingly, while the symmetric model is bounded by the theoretical prediction of $\alpha_c \approx 0.14$, the anti-symmetric variant has a higher capacity - for any finite N, and, presumably, asymptotically.

For continuous dynamics,

$$\dot{x}_i = -x_i + \sum_j W_{ij}\tanh(\beta x_j),$$

the critical capacity of the symmetric Hopfield model [2](with $\mathbf{W}$ symmetric as above) depends on β, the slope of the nonlinearity [5, 6] (which is analogous to temperature in the stochastic discrete model). With $\beta = 2$, the predicted capacity is ≈ 0.07; now, the anti-symmetric variant shows slightly lower capacity than its symmetric counterpart (Supplementary Figure 4B).

3. Model predictions

A central concept in our model is that of imaginary-coded memory. This notion corresponds to properties of network activity as well as connectivity, properties which may be estimated from data recorded during learning and during rest. As discussed in the main text, imaginary-coded memory implies oscillatory motion during retrieval, and furthermore, the strength of representation corresponds to the magnitude of the imaginary part of the coding eigenvalue. This network-level feature manifests as the dominant frequency in the power-spectral density of activity during retrieval (Supplementary Figure 5A, blue). In contrast, no such frequency exists in the population activity at rest (Supplementary Figure 5A, black).

Our model also offers a relation between the strength of a memory trace and the above discussed spectral content: a stronger memory corresponds to a higher dominant frequency during retrieval. To see this, we measure the population-response salience (total signal power) and compare it to the dominant frequency of the response during retrieval of a stored memory. Supplementary Figure 5B shows that, within a single memory item, these two measures are almost perfectly linearly correlated. Tracking the erosion of many memory traces, it is evident that each instance has a slightly different linear dependence (Supplementary Figure 5C); nevertheless, the overall correlation remains significant.

Our model also suggests phenomena that could, in principle, be observed by directly measuring synaptic connections across time. For this analysis, we assume that recorded connections are bidirectional, and that both directions are tracked. As an outcome of imaginary-coded memory, learning should induce strongly anti-correlated fluctuations of reciprocal connections; a homeostasis mechanism which stabilizes learning, by virtue of real-part control, is expected to drive positive correlations.

We test this idea on the two homeostasis mechanisms considered. During rest, in the case of rate-control homeostasis, steady-state fluctuations result in a unimodal zero-mean distribution of correlations (Supplementary Figure 6A top). With decorrelation homeostasis, symmetric-dominant plasticity induces correlated fluctuations (Supplementary Figure 6B top), forming a unimodal distribution with positive mean. The difference between the two homeostasis mechanisms becomes more dramatic during learning. With rate-control, learning drives anti-symmetric synaptic change in the direction of the stimulus, whereas homeostasis acts to stabilize the set-point $\phi = \phi_0$. Since these activity patterns are generally unaligned, the learning and homeostasis terms have distinct eigenspaces, and the result is a bimodal distribution of correlation coefficients (Supplementary Figure 6A bottom).

The picture is qualitatively different with decorrelating homeostasis: during learning, the anti-Hebbian term strongly suppresses the same directions that are learned. This alignment of eigenspaces results in a cancellation between the effects of learning and homeostasis, such that overall, reciprocal connections are positively correlated (Supplementary Figure 6B bottom). Nevertheless, the learning and rest phases can be quantitatively distinguished, as the distribution is shifted towards independence. Finally, after learning the correlations relax back to their respective steady-state distributions (Supplementary Figure 6 top, orange).

These observations suggest that the existence of global subspaces underlying memory can be probed from partial data, specifically by characterizing the statistics of correlations between the

fluctuations of reciprocal connections. A significant difference between learning and rest in these statistics would imply the importance of the imaginary eigenvalues in memory; moreover, the specific form of distributions might provide indirect information on homeostatic mechanisms in the network.

Supplementary Figures

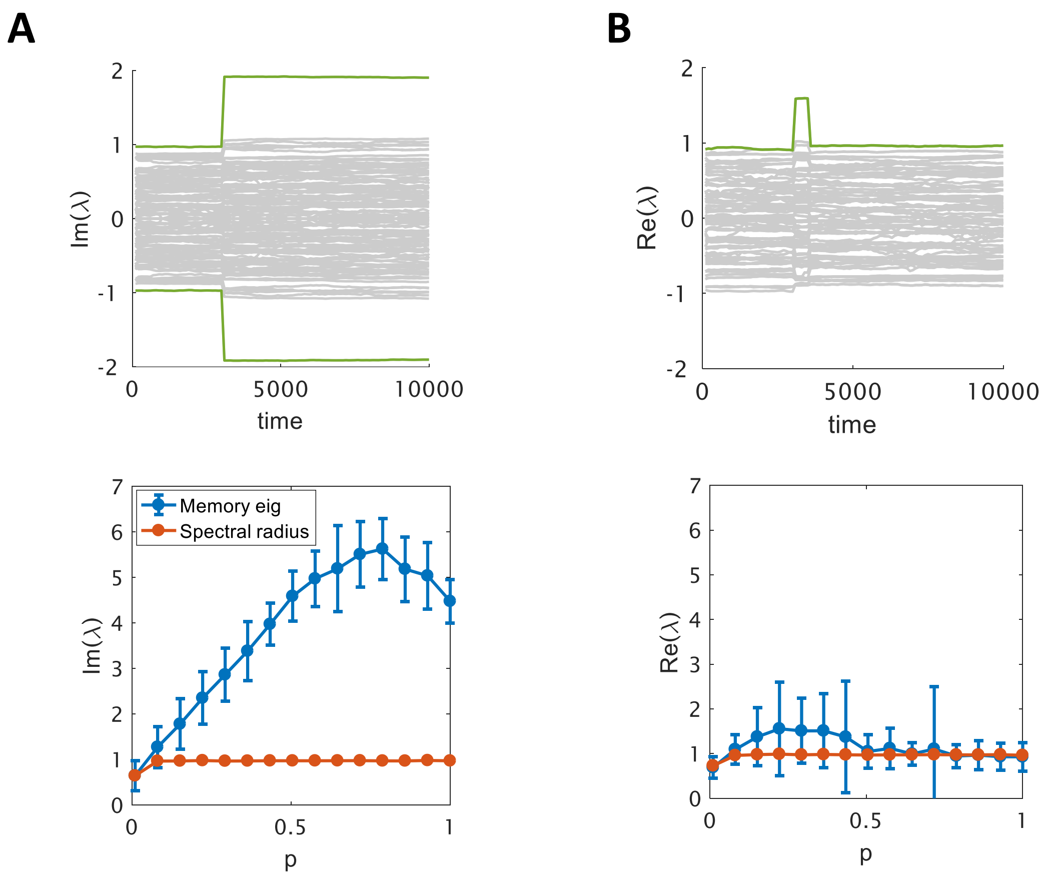

Supplementary Figure 1: Model robustness to sparse connectivity. Networks following the dynamics of Equations (1,2) in the main text, with the rate-control homeostatic rule (Eq. 4 in main text), $N = 128$ neurons, and sparse connectivity are simulated. For each simulation we draw a random adjacency matrix $\mathbf{B} \in \mathbb{R}^{N\times N}$, an Erdos-Renyi graph with connection probability $p \in [0, 1]$ from node j to node i, namely B_{ij} are independent Bernoulli random variables with parameter p. Network connectivity $\mathbf{W}$ is then obtained by multiplying each nonzero connection in $\mathbf{B}$ by an independent Gaussian random variable with mean zero and variance $\frac{1}{pN}$. At time $t = 2500$ we embed a real- or imaginary-coded memory item into the network, as explained in the main text. (A) Imaginary-coded memory. Top: one example simulation, $p = 0.2$, green curve represents the memory eigenvalues. Bottom: mean imaginary magnitude of embedded memory eigenvalues, averaged over 50 network realizations, as a function of the sparseness parameter p. Error bars denote one standard deviation from the mean. Red dots depict the radius of the bulk spectrum. (B) Same as in A, for real-coded memory.

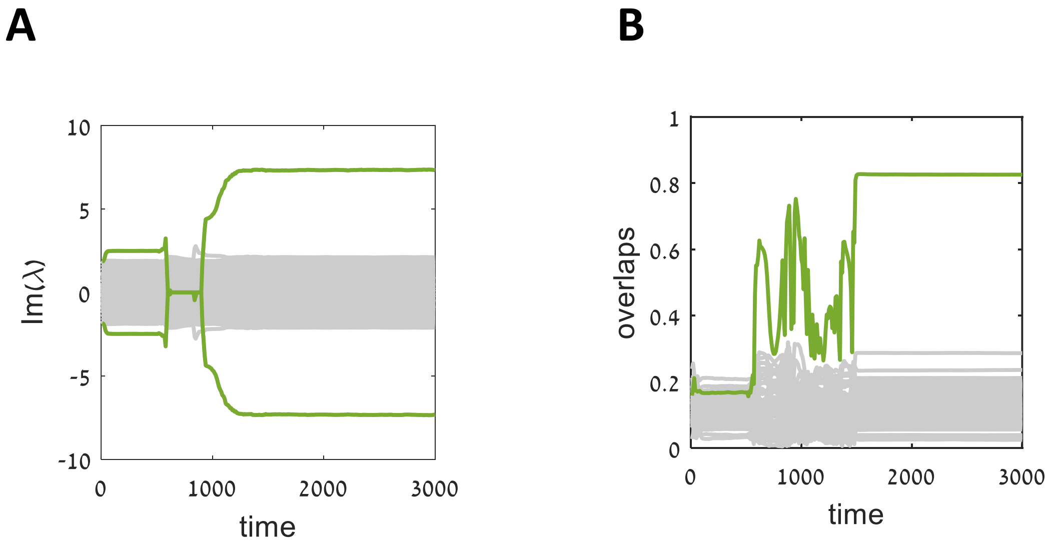

Supplementary Figure 2: Learning with rate-control homeostasis. (A) Imaginary part of the spectrum of the connectivity matrix $\mathbf{W}$ before, during and after external stimulation applied between times $t = 500$ and $t = 600$. Before learning, the imaginary part of the spectrum is almost constant in time. The learning of a memory item manifests as the growth in imaginary amplitude of one complex conjugate eigenvalue pair (green trajectories). (B) During stimulus presentation, the learning rule modifies $\mathbf{W}$ such that the plane spanned by $\mathbf{u}, \mathbf{v}$ is invariant (i.e. $\mathbf{u} \pm i\mathbf{v}$ become eigenvectors of $\mathbf{W}$). Plotted are the overlaps of this eigenplane of $\mathbf{W}$, corresponding to the largest imaginary eigenvalue pair, with $N/2$ planes: the $\mathbf{u}, \mathbf{v}$ (green), and $N/2 - 1$ orthogonal planes (gray); see Methods of main text.

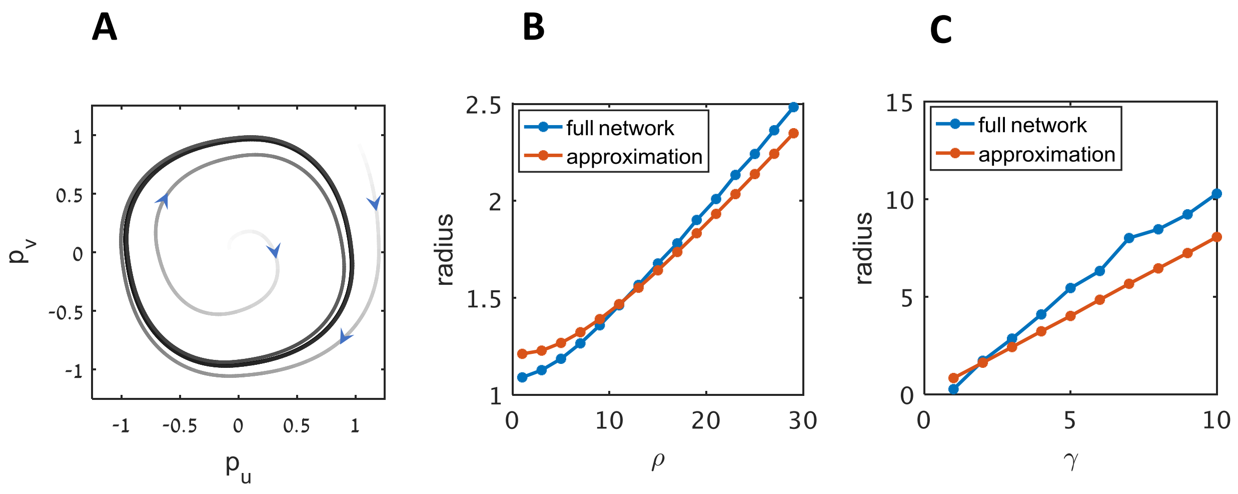

Supplementary Figure 3: Dynamics in the approximate system. (A) Simulation of the approximate system Supplementary Equation (4), which mimics the dynamic behavior of the full system, including the radius of the limit-cycle. Here we use $\rho = 3$ and $\gamma = 1.5$. (B) Comparison of the radii of limit cycles in the full model (blue) and the approximate system (orange) for a range of ρ values; γ was kept fixed at 1.5. (C) Here $\rho = 3$ is fixed, and γ varies. In this case the radius varies over a greater range, and the approximation is less accurate.

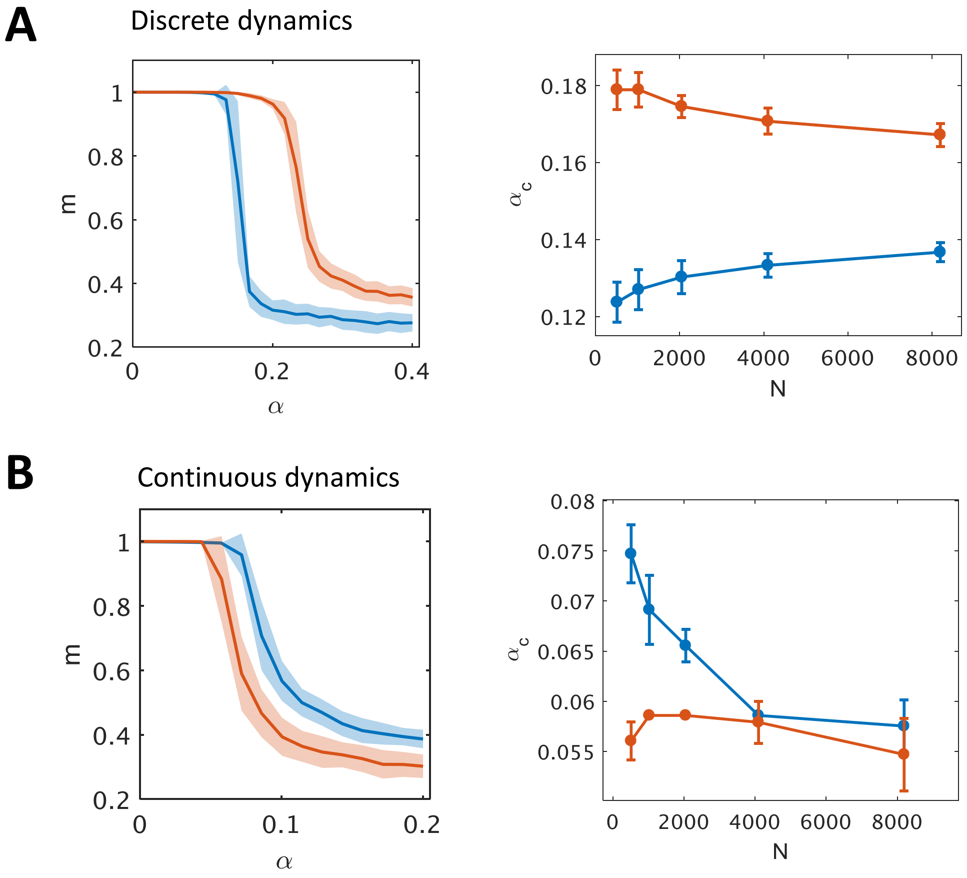

Supplementary Figure 4: Capacity of the Hopfield model. Left: Overlap with a target memory item as a function of memory loading $\alpha = M/N$, for the discrete (A) or continuous (B) models with $N = 4096$ neurons (and $\rho = 8$ in the continuous case); the symmetric model is plotted in blue and the anti-symmetric in orange. Lines represent the final overlap averaged over 100 realizations (each evolved over $T = 50$ time-steps), shaded areas mark one standard deviation from the mean. Right: The critical memory load, the α yielding at least 2% error in overlap, for a range of network sizes N. Data points represent the mean over 20 realizations of the computation in panel A, bars mark one standard deviation from the mean. In all cases we use random patterns with $P(u_i = \pm 1) = 0.5$ independently. In each instance, the network is initiated at a noisy version of the target pattern. For the discrete model, we randomly flip 10% of the bits in the desired target pattern; for the continuous case, a zero-mean Gaussian variable with standard deviation $1/\sqrt{N}$ is added to each component independently.

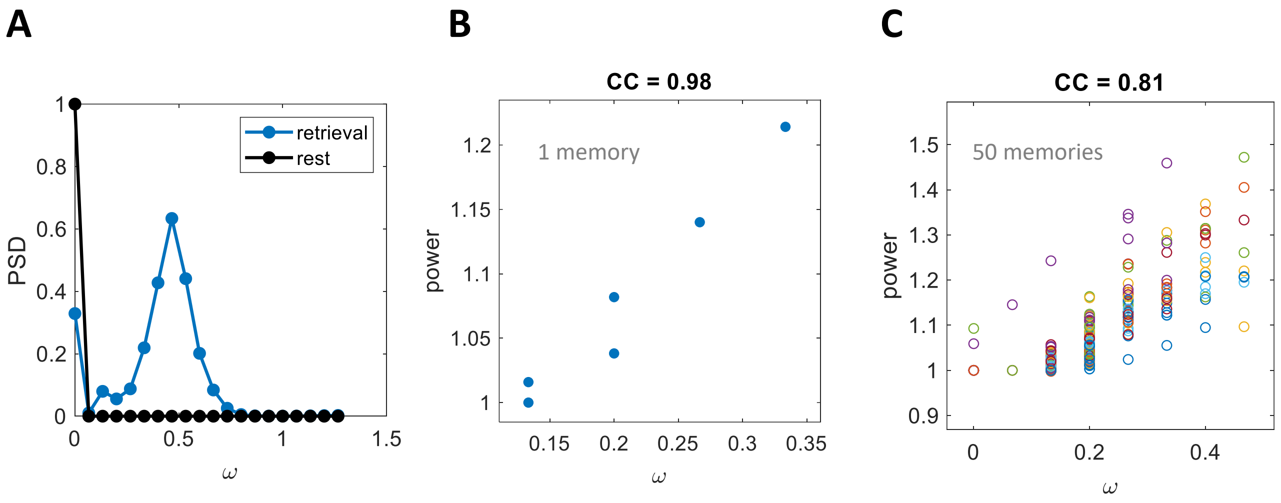

Supplementary Figure 5: Population-level oscillations during memory retrieval. (A) Power-spectral density (PSD) of network activity projected onto one direction on the memory plane ($p_{\mathbf{u}} := \frac{1}{\sqrt{N}}\mathbf{u}^T\mathbf{x}$; see main text) during retrieval (blue) and during rest (black). (B) Normalized energy of the projected coordinate (root-mean-square across time) as a function of the peak frequency in its PSD, during 6 retrieval events of a single stored memory. (C) 50 repeats of the simulation in panel B, wherein each repeat a different memory item is embedded into the same fixed network. In all panels we simulate our model with the rate-control homeostasis rule and $N = 128$.

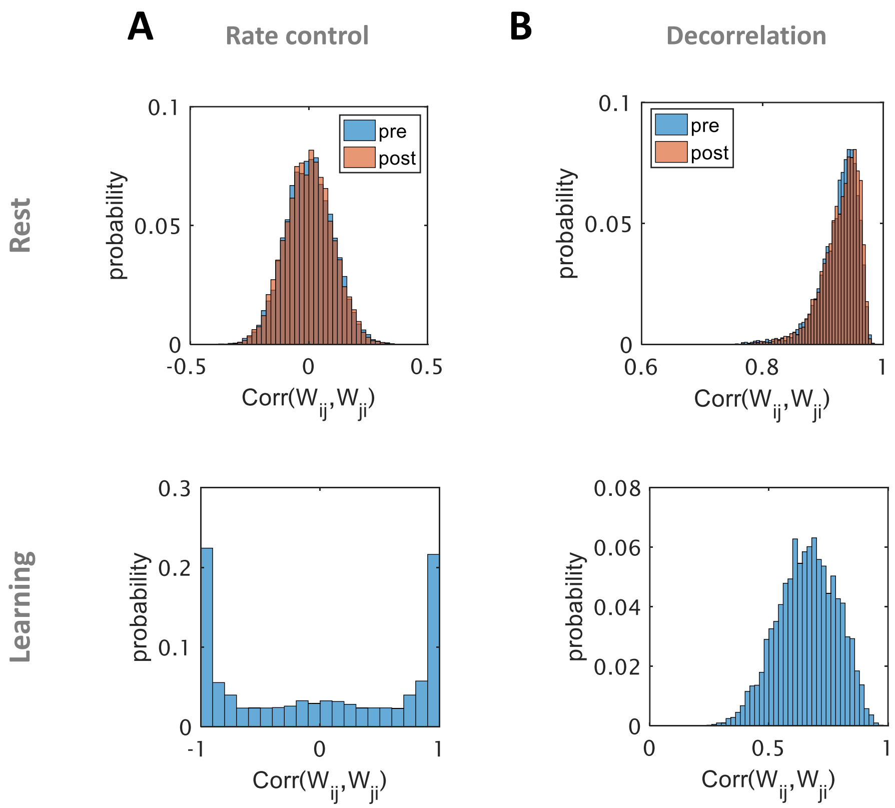

Supplementary Figure 6: Reciprocal fluctuations during learning and rest. Histograms of correlation coefficients between changes of reciprocal connections $\dot{W}_{ij}$ and $\dot{W}_{ji}$, for the rate-control (A) and decorrelation (B) homeostasis mechanisms. Top: correlations at rest, before and after learning. Bottom: correlations during learning. All correlations are computed from a network with $N = 128$ neurons, all other parameters as detailed in Methods.

Supplementary References

[1] J. J. Hopfield. Neural networks and physical systems with emergent collective computational abilities. *Proc. Natl. Acad. Sci. USA*, 79(8):2554–2558, 1982.

[2] J. J. Hopfield. Neurons with graded response have collective computational properties like those of two-state neurons. *Proc. Natl. Acad. Sci. USA*, 81(10):3088–3092, 1984.

[3] Daniel J Amit, Hanoch Gutfreund, and Haim Sompolinsky. Storing infinite numbers of patterns in a spin-glass model of neural networks. *Physical Review Letters*, 55(14):1530, 1985.

[4] E. Goles. Antisymmetrical neural networks. *Discrete Appl. Math.*, 13(1):97–100, 1986.

[5] M. Shiino and T. Fukai. Replica-symmetric theory of nonlinear analogue neural networks. *J. Phys. A: Math. Gen.*, 23(18):L1009, 1990.

[6] R. Kühn, S. Bös, and J. L. van Hemmen. Statistical mechanics for networks of graded-response neurons. *Physical Review A*, 43(4):2084, 1991.
